# Investigating the prognostic and predictive value of the type II cystatin genes in gastric cancer

**DOI:** 10.1186/s12885-023-11550-6

**Published:** 2023-11-17

**Authors:** Ye-yang Chen, Bo-pei Li, Jun-fu Wang, Ye Wang, Shan-shan Luo, Ru-jing Lin, Xi-wen Liao, Jun-Qiang Chen

**Affiliations:** 1 Department of General Surgery, The First People’s Hospital of Yulin, Yulin, China; 2 Department of Gastrointestinal Surgery, The First Affiliated Hospital of Guangxi Medical University, Nanning, China; 3 Department of General Surgery, The First Affiliated Hospital of Nanchang University, Nanchang, China; 4 Department of Colorectal Surgery, Affiliated Tumor Hospital of Guangxi Medical University, Nanning, China; 5 Department of General Surgery, The People’s Hospital of Binyang, Nanning, China; 6 Department of Hepatobiliary Surgery, The First Affiliated Hospital of Guangxi Medical University, Nanning, China

**Keywords:** Cystatins (CST), The Cancer Genome Atlas (TCGA), Gastric cancer (GC), Prognosis, Biomarker

## Abstract

**Background:**

Accumulating evidence indicates that type II cystatin (CST) genes play a pivotal role in several tumor pathological processes, thereby affecting all stages of tumorigenesis and tumor development. However, the prognostic and predictive value of type II CST genes in GC has not yet been investigated.

**Methods:**

The present study evaluated the expression and prognostic value of type II CST genes in GC by using The Cancer Genome Atlas (TCGA) database and the Kaplan–Meier plotter (KM plotter) online database. The type II CST genes related to the prognosis of GC were then screened out. We then validated the expression and prognostic value of these genes by immunohistochemistry. We also used Database for Annotation, Visualization, and Integrated Discovery (DAVID), Gene Multiple Association Network Integration Algorithm (GeneMANIA), Search Tool for the Retrieval of Interacting Genes/Proteins (STRING), nomogram, genome-wide co-expression analysis, and other bioinformatics tools to analyze the value of type II CST genes in GC and the underlying mechanism.

**Results:**

The data from the TCGA database and the KM plotter online database showed that high expression of CST2 and CST4 was associated with the overall survival (OS) of patients with GC. The immunohistochemical expression analysis showed that patients with high expression of CST4 in GC tissues have a shorter OS than those with low expression of CST4 (HR = 1.85,95%CI: 1.13–3.03, *P* = 0.015). Multivariate Cox regression analysis confirmed that the high expression level of CST4 was an independent prognostic risk factor for OS.

**Conclusions:**

Our findings suggest that CST4 could serve as a tumor marker that affects the prognosis of GC and could be considered as a potential therapeutic target for GC.

**Supplementary Information:**

The online version contains supplementary material available at 10.1186/s12885-023-11550-6.

## Introduction

Globally, gastric cancer (GC) is the fifth most common malignant tumor in new incidence and the fourth leading cause of cancer-related death worldwide [1]. The incidence of GC is highly region-specific, with the highest incidence in East Asia, including China [2]. The 2015 statistics from the National Cancer Center in China showed that there were 679,000 new cases of GC and 498,000 deaths from GC in China, and the new incidence of morbidity and mortality due to GC ranked the second among all malignant tumors in China during the same period [3]. According to statistics, the 5-year overall survival (OS) rate of patients with GC in China in 2015 was 35.1%, while the 5-year OS rate of patients with GC in the United States in 2014 was only 33.1% [4, 5]. The reason for this low survival rate is that only a small number of patients with GC are diagnosed at an early stage, and most patients with GC are diagnosed at an advanced stage; moreover, there are only few effective treatment approaches for patients in advanced stage [6]. Therefore, it is critical to explore new molecular biomarkers for the diagnosis, treatment, and prognosis of GC.

The cystatin (CST) superfamily are a kind of thiol proteinase inhibitors (TPIs) that are widely present in human tissues and body fluids and function as competitive and reversible inhibitors of cysteine proteases. The CST superfamily is divided into three subfamilies according to their three-dimensional molecular structure and biochemical function [7]: (1) type I CSTs are also known as stefins and include stefin A and stefin B [8]; (2) type II CSTs are extracellular proteins; their members include CST C, CST D, CST E/M, CST F, CST S, CST SN, and CST SA, and their coding genes are CST3, CST5, CST6, CST7, CST4, CST1, and CST2, respectively [9]; and (3) type III CSTs, also known as kininogens, are multidomain proteins. There are three types of kininogens: high-molecular-weight kininogens, low-molecular-weight kininogens, and T-type kininogens [10]. Several studies have shown that type II CSTs play a pivotal role in many pathological processes and affect various stages of tumorigenesis and tumor development, including proliferation, apoptosis, invasion, metastasis, and angiogenesis [11–13]. To date, no relevant research studies have been conducted on the expression and prognostic value of type II CST genes in GC.

To determine the prognostic value and potential function of type II CST genes in GC, we analyzed the expression and prognostic value of type II CST genes by using The Cancer Genome Atlas (TCGA; https://portal.gdc.cancer.gov) database and the Kaplan–Meier plotter (KM plotter) online database. We also used immunohistochemistry to validate the expression of type II CST genes in GC tissues and their prognostic value. Finally, various bioinformatics tools were used to determine the potential function and mechanism of type II CST genes.

## Materials and methods

### Functional and co‑expression analyses

The Database for Annotation, Visualization, and Integrated Discovery (DAVID, v.6.8; https://david.ncifcrf.gov; accessed September 1, 2020) was used to analyze Gene Ontology (GO) and Kyoto Encyclopedia of Genes and Genomes (KEGG) enrichment of type II CST genes [14, 15]. The Gene Multiple Association Network Integration Algorithm (GeneMANIA v.3.6.0; http://www.genemania.org/; accessed May 20, 2019) was used to predict the function of type II CST genes [16]. The Search Tool for the Retrieval of Interacting Genes (STRING v.11.0; https://string-db.org/; accessed May 20, 2019) was used to detect protein–protein interaction (PPI) networks [17].

### TCGA

We downloaded mRNA expression data and the corresponding clinical information related to GC from the TCGA (https://portal.gdc.cancer.gov; accessed August 22, 2018). The TCGA database included 407 patients diagnosed with GC, including 375 GC tissues and 32 adjacent normal tissues. After excluding cases with missing follow-up data, 351 patients with GC were included in our present analysis. We used the TCGA database to perform paired t-test on the mRNA expression levels of type II CST genes in GC and adjacent normal tissues, and scatter plots and receiver operating characteristic (ROC) curves were then plotted. For survival analysis, patients with GC were divided into two groups on the basis of the median expression level of type II CST genes: high expression group and low expression group. The mRNA expression of type II CST genes and clinical information in the TCGA database were used for the univariate survival analysis and the multivariate Cox regression model survival analysis.

### KM plotter online database

The KM plotter online database (http://kmplot.com/analysis/index.php?p=service&cancer=gastric; accessed September 27, 2020) was used to analyze the relationship between the mRNA expression of type II CST genes and the OS of patients with GC. The KM plotter online database was established using the gene expression data and survival information of 875 GC patients; these data were downloaded from the Gene Expression Omnibus (GEO) (GSE14210, GSE15459, GSE22377, GSE29272, GSE51105, and GSE62254) [18]. The KM plotter online database is used for the discovery and verification of biomarkers. The members of the type II CST family were entered into the KM plotter online database for analysis. All GC patients were divided into two groups on the basis of the median mRNA expression level of type II CST genes: high expression group and low expression group. Finally, parameters such as survival analysis graph, hazard ratio (HR), 95% confidence interval (CI), and log-rank *P* were analyzed.

### Nomogram

A nomogram was constructed, and the prognostic risk between type II CST genes and the OS grade in patients with GC was assessed. The possible utility of type II CST genes to predict clinical rank was also assessed. According to prognostic clinical indicators and Cox regression model survival analysis, related factors and type II CST gene expression levels were included in the risk model. The scores for each factor were counted, and the 1-, 3-, 5-, and 10-year survival rates were calculated [19]. The nomogram was constructed using the rms package (https://CRAN.R-project.org/package=rms), along with its dependencies in the R platform, and visualized using gplot [20].

### Stratified analyses

The stratified analysis method was used to evaluate the prognostic value of type II CST genes in different stratifications in the TCGA and the KM plotter online databases.

### Immunohistochemistry

GC sections with complete clinicopathological and prognostic data were collected from the Affiliated Hospital of Guangxi Medical University. The sections were placed in 65◦C oven for 2 h, dewaxed with xylene, hydrated with a graded series of ethanol, and repaired with antigen repair buffer by EDTA method. Endogenous antigens were blocked with 3% hydrogen peroxide. The sections were incubated with CST2 antibody (Thermo Fisher Scientific, 1:150 dilution) and CST4 antibody (ABclonal, 1:200 dilution) overnight at 4◦C. The sections were heated for 30 min to room temperature and incubated with secondary antibody for 20 min. The staining result was visualized using DAB color developing solution. The sections were stained with Hematoxylin, differentiated in 1% hydrochloric acid in alcohol, and dehydrated. Thereafter, the sections were naturally dried in a fume hood, transparentized by xylene, and sealed with neutral resin. The negative control used PBS instead of primary antibody, and the positive section was served as positive control. Immunohistochemistry was performed to determine the expression of type II CST genes in GC and adjacent normal tissues. Paired t-test was used to analyze the difference in the expression level between GC and adjacent normal tissues, and scatter plots and ROC curves were drawn. The X-tile software was used to determine the optimal cutoff value. X-tile, a statistical software developed by Yale University with a single function, can determine the cut-off point of continuous variables and facilitate the drawing of the Kaplan–Meier curve [21]. Finally, the univariate survival analysis and multivariate Cox regression model survival analysis of immunohistochemical expression were performed to analyze the prognosis of patients with GC who were positive or negative for type II CST genes.

### Genome-wide co-expression analysis and functional enrichment of type II CST prognostic genes

Pearson’s correlation analysis was performed between type II CST prognostic genes and TCGA genome expression profile data, and the co-expressed genes were screened out. Pearson’s correlation coefficient was calculated by the cor function on the R 3.4.4 platform. The difference was considered to be significant at |r|> 0.6,* P* < 0.05. These co-expressed genes were then used to perform GO and KEGG functional enrichment analyses by using DAVID v6.8 [22–24].

### Statistical analyses

Statistical analysis was performed using SPSS v.25.0 software (IBM Corp., Armonk, NY, USA) and GraphPad Prism software (GraphPad Software, Inc., La Jolla, CA, USA) to generate vertical scatterplots and survival curves. Nomograms and correlations were also generated using R software (Vienna, Austria).

## Results

### Functional enrichment of type II CST genes and co-expression analysis

**Fig. 1:**
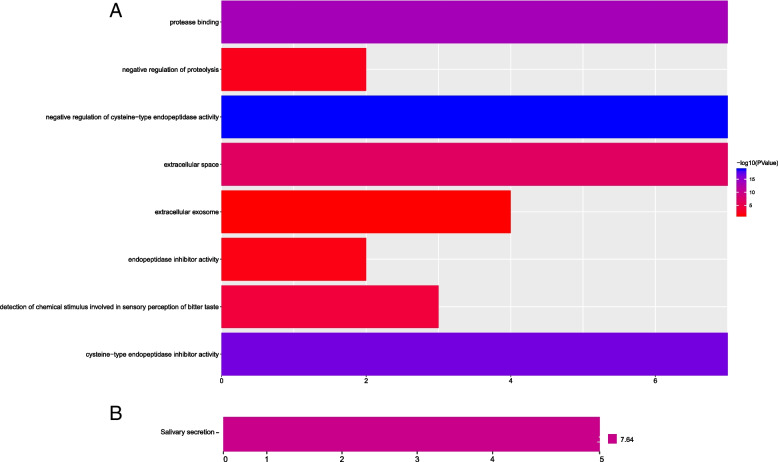
GO and KEGG analysis. (A)GO analysis of Type II CST genes. (B)KEGG analysis of Type II CST genes. CST, Cystatins; GO, Gene Ontology; KEGG, Kyoto Encyclopedia of Genes and Genomes

**Fig. 2:**
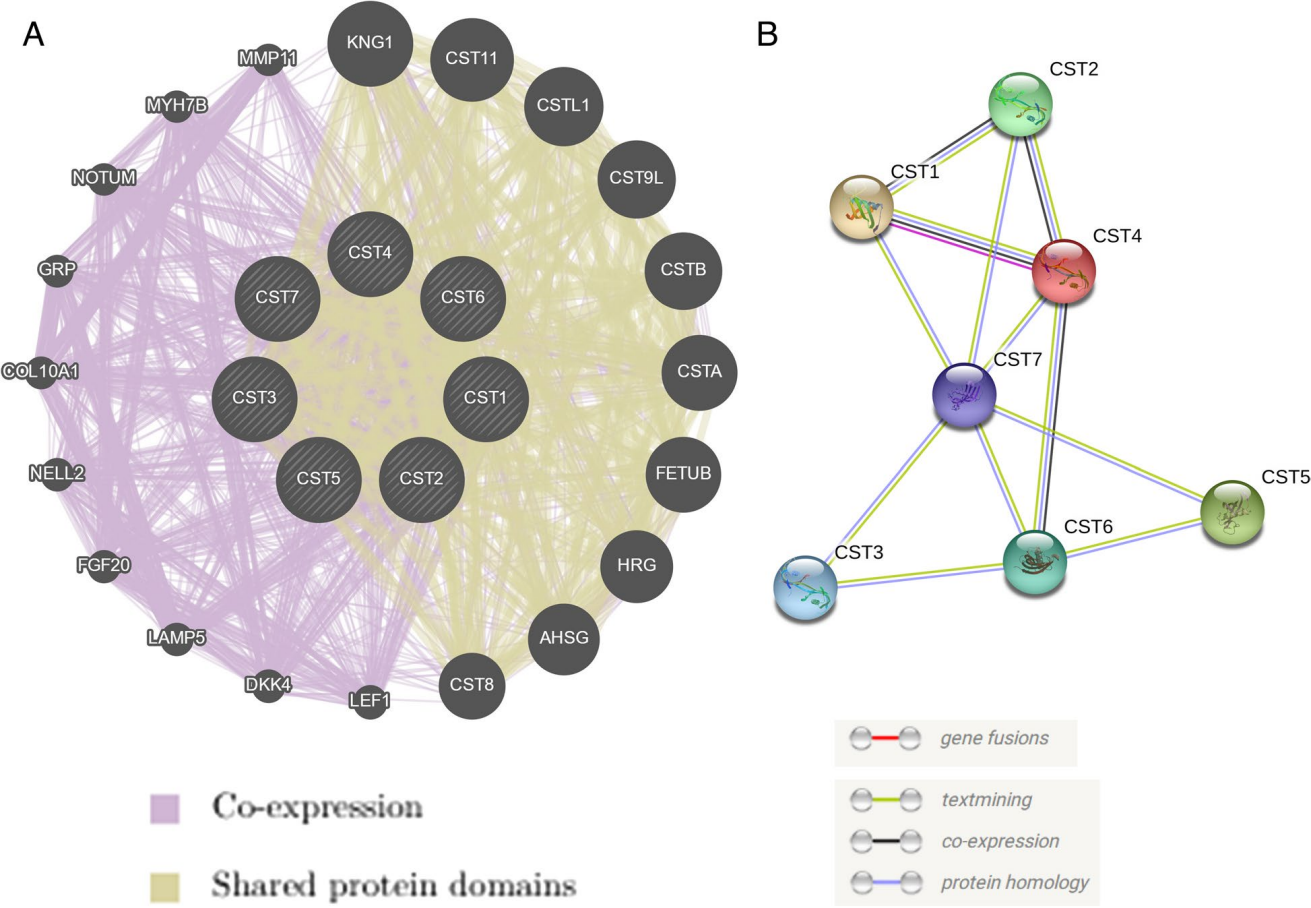
Gene and protein interaction networks. **A** Type II CST genes multiple association network integration algorithm. The size of the circle represents the strength of the co‑expression. **B** Protein‑protein interaction networks. CST, Cystatins

The biological function of type II CST genes was assessed by DAVID, and biological process (BP), cellular component (CC), and molecular functionality (MF) were obtained from GO and KEGG pathway analyses (Fig. 1A and B). GO analysis showed that the type II CST gene family was enriched in protease binding, negative regulation of proteolysis, negative regulation of cysteine-type endopeptidase activity, extracellular space, extracellular exosome, endopeptidase inhibitor activity, and cysteine-type endopeptidase inhibitor activity (Fig. 1A). KEGG pathway analysis showed that the type II CST gene family was enriched in salivary secretion (Fig. 1B). The gene–gene interaction network of type II CST genes is shown in Fig. 2A; the figure shows that the members of the type II CST gene family co-express and interact with other genes. According to the information in the STRING database, the PPI interaction network showed direct or indirect connections between members of the type II CST gene family, among which CST7 was directly related to other family members (Fig. 2B).

### Survival analysis in the TCGA

**Fig. 3:**
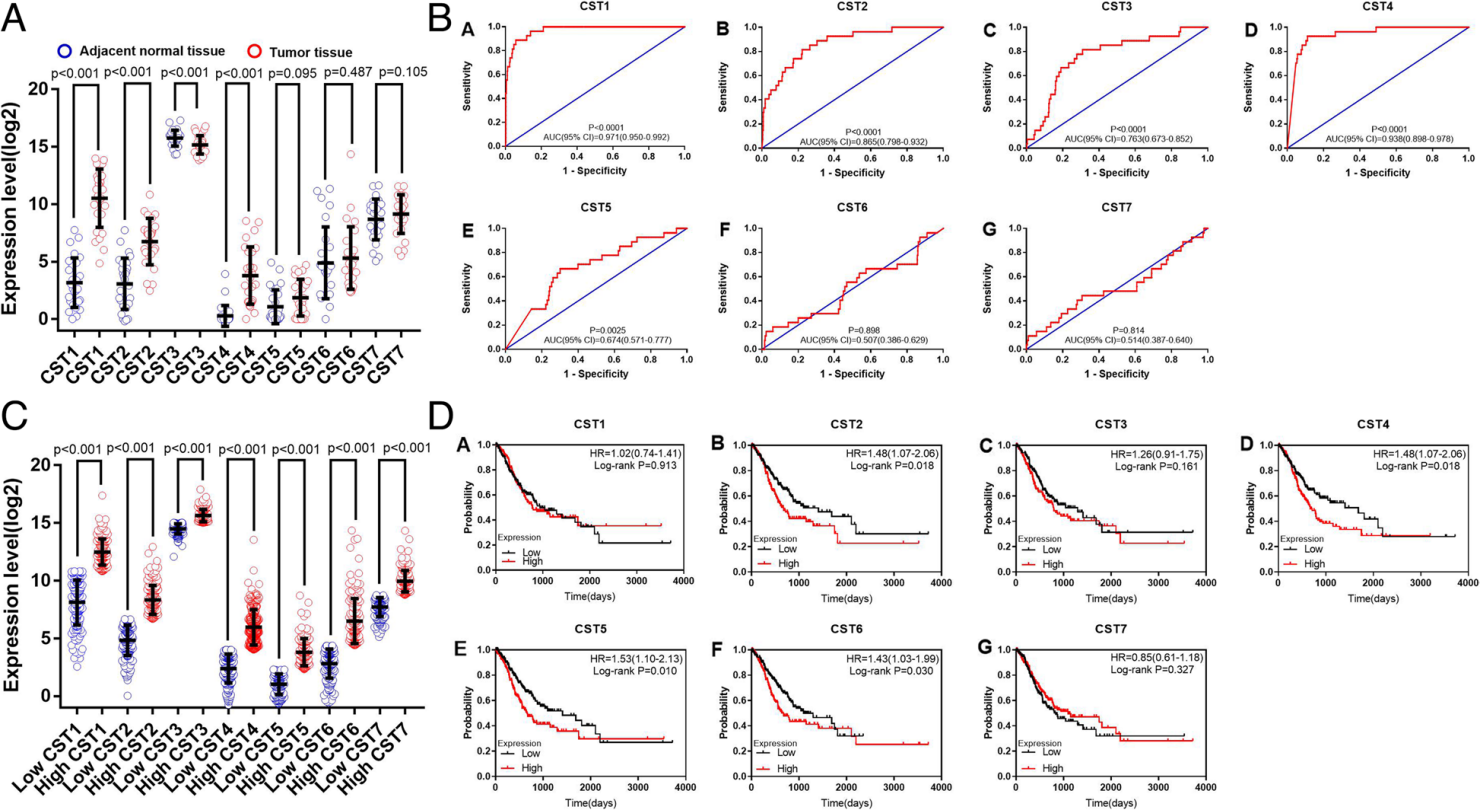
Survival analysis in The Cancer Genome Atlas. **A** Scatterplots for Type II CST genes expression levels in The Cancer Genome Atlas. **B** ROC curve of the expression level of type II CST genes in The Cancer Genome Atlas. **C** Scatterplots for Type II CST genes expression levels in The Cancer Genome Atlas. Red, high expression; blue, low expression. **D** Prognostic graphs illustrating the impact of Type II CST genes expression on overall survival in The Cancer Genome Atlas. Kaplan‑Meier survival curves for patients with gastric cancer according to median expression of CST1-7 (*n* = 351). CST, Cystatins; HR, hazard ratio (95% CI)

From the vertical scatter plot of type II CST gene expression levels in the TCGA, it was observed that the expression levels of CST1, CST2, CST3, and CST4 were significantly different in GC tissues and adjacent normal tissues (*P* < 0.05, Fig. 3A). The ROC curve of the expression level of type II CST genes in the TCGA showed that the expression levels of the genes CST1, CST2, CST3, CST4, and CST5 were significantly different (*P* < 0.001, Fig. 3B). The vertical scatter plot of the expression levels of type II CST genes in the TCGA revealed a significant difference between the high and low expression of all type II CST genes (*P* < 0.001, Fig. 3C). Figure 3D shows the results of the univariate survival analysis of the expression levels of type II CST genes in the TCGA. The high expression level of CST2, CST4, CST5, and CST6 in patients with GC was significantly correlated with poor prognosis (*P* = 0.018, HR = 1.48, 95% CI = 1.07–2.06; *P* = 0.018, HR = 1.48, 95% CI = 1.07–2.06; *P* = 0.010, HR = 1.53, 95% CI = 1.10–2.13; *P* = 0.030, HR = 1.43, 95% CI = 1.03–1.99, respectively).

Univariate survival analysis was performed to explore the prognostic value of clinical factors in patients with GC, including gender, age, tumor location, Helicobacter pylori infection, histological type, histologic grade, microsatellite stability (MMS), tumor stage, cancer status, residual tumor, chemotherapy, and targeted therapy. The results showed that age, tumor stage, cancer status, residual tumor, chemotherapy, and targeted therapy were associated with OS (all *P* < 0.05, Supplemental Table S1). After including age, tumor stage, cancer status, residual tumor, chemotherapy, and targeted therapy as adjustment factors in the Cox proportional hazards regression models, the results of the multivariate survival analysis showed that high expression of the genes CST2, CST4, and CST5 significantly decreased OS (adjusted *P* = 0.030, HR = 1.57, 95% CI = 1.05–2.35; adjusted *P* = 0.010, HR = 1.69, 95% CI = 1.13–2.51; adjusted *P* = 0.001, HR = 1.94, 95% CI = 1.29–2.91, respectively; Table 1). This result was consistent with that of the univariate survival analysis (*P* = 0.018, HR = 1.48, 95% CI = 1.07–2.06; *P* = 0.018, HR = 1.48, 95% CI = 1.07–2.06; *P* = 0.010, HR = 1.53, 95% CI = 1.10–2.13, respectively; Table 1; Fig. 3D). However, the expression level of the gene CST6 did not show a significant difference in the multivariate survival analysis (*P* = 0.515, HR = 1.15, 95% CI = 0.76–1.72; Table 1), which was inconsistent with the results of the univariate survival analysis (*P* = 0.030, HR = 1.43, 95% CI = 1.03–1.99; Table 1; Fig. 3D).

**Table 1:** Analysis of the association between CST genes and the risk of death in The Cancer Genome Atlas gastric cancer cohort (*n* = 351)

**Gene expression status**	**Patients (*n* = 351)**	**Events, n (%)**	**MST (days)**	**Crude HR (95% CI)**	**Crude *P*-value**	**Adjusted HR**^**a**^** (95% CI)**	**Adjusted *P*-value**^**a**^
**CST1**					0.913		0.094
Low	176	73 (41.5)	940	Ref.		Ref.	
High	175	71 (40.6)	782	1.02 (0.74–1.41)		1.42 (0.94–2.13)	
**CST2**					**0.018**		**0.030**
Low	176	66 (37.5)	1407	Ref.		Ref.	
High	175	78 (44.6)	669	1.48 (1.07–2.06)		1.57 (1.05–2.35)	
**CST3**					0.161		0.130
Low	176	69 (39.2)	1294	Ref.		Ref.	
High	175	75 (42.9)	792	1.26 (0.91–1.75)		1.37 (0.91–2.05)	
**CST4**					**0.018**		**0.010**
Low	176	63 (35.8)	1686	Ref.		Ref.	
High	175	81 (46.3)	675	1.48 (1.07–2.06)		1.69 (1.13–2.51)	
**CST5**					**0.010**		**0.001**
Low	176	64 (36.4)	1407	Ref.		Ref.	
High	175	80 (45.7)	661	1.53 (1.10–2.13)		1.94 (1.29–2.91)	
**CST6**					0.030		0.515
Low	176	66 (37.5)	1095	Ref.		Ref.	
High	175	78 (44.6)	669	1.43 (1.03–1.99)		1.15 (0.76–1.72)	
**CST7**					0.327		0.284
Low	176	73 (41.5)	832	Ref.		Ref.	
High	175	71 (40.6)	1095	0.85 (0.61–1.18)		0.81 (0.54–1.20)	

*Abbreviations*: *GC* Gastric cancer, *CST* Cystatins, *MST* Median survival time, *HR* Hazard ratio, *CI* Confidence interval

^a^Adjusted for age, tumor stage, cancer status, residual tumor, chemotherapy, and targeted therapy. Bold figures indicate statistically significance

### Validation of the CST cohort using the KM plotter online database

**Fig. 4:**
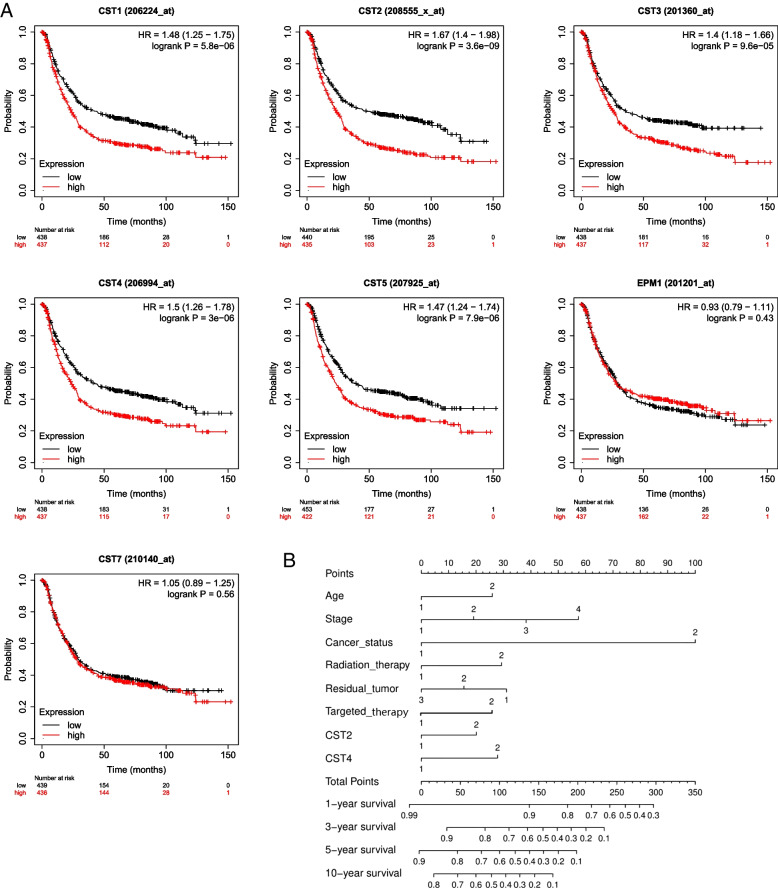
Survival analysis in the Kaplan-Meier plotter online database and Nomogram analysis. **A** Prognostic graphs of Type II CST median expression for overall survival generated using the Kaplan-Meier plotter online database. **B** Nomogram for the association between clinicopathological data and risk score. CST, Cystatins; HR, hazard ratio (95% CI)

The expression and prognostic value of type II CST genes were validated using the KM plotter online database. The KM curves of type II CST genes are shown in Fig. 4A. The analysis results showed that GC patients with high mRNA levels of CST1, CST2, CST3, CST4, CST5, and CST6 had significantly worse OS (*P* = 5.8e-06, HR = 1.48, 95% CI = 1.25–1.75; *P* = 3.6e-09, HR = 1.67, 95% CI = 1.40–1.98; *P* = 9.6e-05, HR = 1.40, 95% CI = 1.18–1.66; P = 3e-06, HR = 1.50, 95% CI = 1.26–1.78; *P* = 7.9e-06, HR = 1.47, 95% CI = 1.24–1.74; *P* < 0.001, HR = 1.40, 95% CI = 1.18–1.66, respectively). Consistent with the TCGA analysis, the expression of CST2, CST4, and CST5 was significantly associated with worse prognosis (adjusted *P* = 0.030, HR = 1.57, 95% CI = 1.05–2.35; adjusted *P* = 0.010, HR = 1.69, 95% CI = 1.13–2.51; adjusted *P* = 0.001, HR = 1.94, 95% CI = 1.29–2.91, respectively; Table 1). Therefore, we screened out CST2 and CST4 for further analyses.

### Nomogram and stratified analysis

Nomograms were drawn based on CST2 and CST4 expression levels, age, tumor stage, cancer status, residual tumor, and targeted therapy. Next, the 1-, 3-, 5-, and 10-year survival rates were calculated (Fig. 4B). The results of nomogram analysis revealed that CST2 and CST4 had certain predictive effects on the prognosis of patients with GC. In particular, the predictive effect of CST4 was higher than that of age and targeted therapy.

Stratified analysis was used to analyze the prognostic value of the CST2 and CST4 genes in GC, and the relationship between CST2 and CST4 and OS was analyzed using the TCGA database (Tables 2 and 3) and the KM plotter online database (Tables 4 and 5). As shown in Table 2, high levels of CST2 mRNA were significantly associated with poor prognosis in the histological signet ring type, tumor stage IV, residual tumor R0, radiotherapy, and target therapy in the TCGA. As noted in Table 3, high levels of CST4 mRNA were significantly associated with poor prognosis in female, age over 65 years, histologic grade G2 and G3, cancer status as with tumor, residual tumor R0 and R1, radiotherapy, and target therapy in the TCGA. As shown in Table 4, high CST2 mRNA levels showed significantly worse prognosis in male and female, tumor stage III and IV, Lauren classification as intestinal and diffuse, poorly differentiated tumor, surgery alone as treatment, and positive and negative HER2 status in the KM plotter online database. As noted in Table 5, high CST4 mRNA levels showed significantly worse prognosis in male and female, tumor stage III and IV, Lauren classification as intestinal and diffuse, well-differentiated tumor, surgery alone as treatment, and positive and negative HER2 status in the KM plotter online database.

**Table 2:** Stratified analysis of association between CST2 and overall survival in TCGA GC cohort

**Variables**	**Total (*n* = 351)**	**Low CST2 (n)**	**High CST2 (n)**	**HR (95%CI)**	**Log-rank *P*-value**
**Gender**					
Male	226	113	113	1.26 (0.85–1.86)	0.249
Female	125	63	62	1.59 (0.87–2.88)	0.122
**Age (years)**					
< 60	108	54	54	1.50 (0.78–2.89)	0.220
≥ 60	240	120	120	1.27 (0.87–1.85)	0.219
Missing	3				
Location					
Gastroesophageal junction	84	42	42	1.60 (0.83–3.10)	0.154
Body/fundus	123	62	61	1.71 (0.98–2.98)	0.057
Antrum	130	65	65	1.19 (0.69–2.06)	0.052
Missing	14				
Hp infection					
Positive	18	9	9	1.31 (0.26–6.60)	0.735
Negative	143	72	71	1.46 (0.88–2.42)	0.142
Missing	190				
Histological_type					
Intestinal	160	80	80	1.46 (0.89–2.41)	0.121
Diffuse Type	61	31	30	1.10 (0.49–2.44)	0.820
Signet Ring Type	11	6	5	5.2 (0.79–34.09)	**0.005**
Other	118	59	59	1.05 (0.60–1.85)	0.862
Missing	1				
**Histologic grade**					
**G1**	9	5	4	4.33 (0.12–151.0)	0.197
**G2**	127	64	63	1.21 (0.69–2.14)	0.505
**G3**	206	103	103	1.34 (0.88–2.02)	0.171
Missing	9				
**MMS**					
MSI-H	240	120	120	1.41 (0.95–2.10)	0.084
MSI-L	51	26	25	1.30 (0.56–2.99)	0.542
MMS	59	30	29	1.16 (0.51–2.63)	0.722
Missing	1				
Tumor stage					
I	47	24	23	1.83 (0.56–5.96)	0.328
II	109	55	54	1.78 (0.91–3.51)	0.091
III	147	74	73	1.26 (0.79–2.03)	0.331
IV	35	18	17	1.01 (0.43–2.37)	**0.001**
Missing	13				
Cancer status					
Tumor free	206	103	103	1.59 (0.82–3.09)	0.171
With tumor	118	59	59	1.39 (0.91–2.14)	0.117
Missing	27				
Residual_tumor					
R1	14	7	7	1.13 (0.30–4.22)	0.856
R2	14	7	7	0.76 (0.25–2.36)	0.617
R0	287	144	143	1.76 (1.19–2.62)	**0.004**
Missing	36				
Radiotherapy					
Yes	62	31	31	3.17 (1.29–7.79)	**0.019**
No	266	133	133	1.27 (0.88–1.82)	0.199
Missing	23				
Targeted Therapy					
Yes	151	76	75	2.09 (1.23–3.56)	**0.005**
No	175	88	87	1.02 (0.66–1.59)	**0.010**
Missing	25				

Bold figures indicate statistically significance

*Abbreviations*: *CST* Cystatins, *GC* Gastric cancer, *HR* Hazard ratio, *CI* Confidence interval

**Table 3:** Stratified analysis of association between CST4 and overall survival in TCGA GC cohort

**Variables**	**Total (*n* = 351)**	**Low CST2 (n)**	**High CST2 (n)**	**HR (95%CI)**	**Log-rank *P*-value**
**Gender**					
Male	226	113	113	1.16 (0.78–1.71)	0.461
Female	125	63	62	2.33 (1.28–4.27)	**0.005**
**Age (years)**					
< 60	108	54	54	1.31 (0.68–2.52)	0.661
≥ 60	240	120	120	1.51 (1.03–2.20)	**0.034**
Missing	3				
Location					
Gastroesophageal junction	84	42	42	1.15 (0.60–2.22)	0.669
Body/fundus	123	62	61	1.48 (0.85–2.58)	0.167
Antrum	130	65	65	1.45 (0.84–2.50)	0.176
Missing	14				
Hp infection					
Positive	18	9	9	1.06 (0.21–5.27)	0.940
Negative	143	72	71	1.62 (0.97–2.68)	0.064
Missing	190				
Histological_type					
Intestinal	160	80	80	1.58 (0.96–2.58)	0.065
Diffuse Type	61	31	30	1.89 (0.85–4.22)	0.119
Signet Ring Type	11	6	5	1.54 (0.37–6.35)	0.484
Other	118	59	59	1.49 (0.85–2.62)	0.170
Missing	1				
**Histologic grade**					
**G1**	9	5	4	0.20 (0.00–11.57)	0.439
**G2**	127	64	63	1.76 (1.00–3.11)	**0.046**
**G3**	206	103	103	1.53 (1.02–2.32)	**0.044**
Missing	9				
**MMS**					
MSI-H	240	120	120	1.40 (0.94–2.08)	0.090
MSI-L	51	26	25	2.14 (0.92–4.95)	0.078
MMS	59	30	29	1.56 (0.69–3.55)	0.281
Missing	1				
Tumor stage					
I	47	24	23	2.68 (0.80–8.99)	0.084
II	109	55	54	1.69 (0.86–3.34)	0.122
III	147	74	73	1.42 (0.88–2.27)	0.149
IV	35	18	17	0.81 (0.35–1.86)	0.613
Missing	13				
Cancer status					
Tumor free	206	103	103	1.21 (0.62–2.35)	0.573
With tumor	118	59	59	1.71 (1.11–2.62)	**0.010**
Missing	27				
Residual_tumor					
R1	14	7	7	6.00 (1.55–23.24)	**0.009**
R2	14	7	7	0.45 (0.14–1.45)	0.149
R0	287	144	143	1.61 (1.09–2.38)	**0.018**
Missing	36				
Radiotherapy					
Yes	62	31	31	3.50 (1.42–8.65)	**0.010**
No	266	133	133	1.37 (0.95–1.98)	0.087
Missing	23				
Target Therapy					
Yes	151	76	75	2.15 (1.27–3.63)	**0.005**
No	175	88	87	1.19 (0.76–1.85)	0.450
Missing	25				

Bold figures indicate statistically significance

*Abbreviations*: *CST* Cystatins, *GC* Gastric cancer, *HR* Hazard ratio, *CI* Confidence interval

**Table 4:** Stratified analysis of association between CST2 and overall survival in KM plotter GC cohort

**Variables**	**Patients (*n* = 875)**	**HR (95%CI)**	**Log-rank *P*-value**
**Gender**			
Female	236	2.04 (1.43–2.91)	**6.3e-05**
Male	544	1.64 (1.32–2.03)	**5.9e-06**
Missing	95		
**Stage**			
I	67	1.52 (0.56–4.11)	0.400
II	140	1.33 (0.72–2.45)	0.360
III	305	1.61 (1.21–2.15)	**0.001**
IV	148	1.59 (1.08–2.34)	**0.017**
Missing	215		
**Lauren classification**			
Intestinal	320	2.08 (1.51–2.87)	**5.8e-06**
Diffuse	241	1.60 (1.13–2.25)	**0.007**
Mixed	32	1.44 (0.51–4.01)	0.490
Missing	282		
**Differentiation**			
Poorly	165	1.50 (1.01–2.24)	**0.044**
Moderately	67	1.76 (0.91–3.41)	0.092
Well	32	2.32 (0.95–5.65)	0.056
Missing	611		
**Treatment**			
Surgery alone	380	1.36 (1.02–1.82)	**0.035**
5-Fu based adjuvant	152	1.01 (0.72–1.43)	0.940
Other adjuvant	76	0.69 (0.28–1.69)	0.410
Missing	267		
**HER2 status**			
Negative	532	1.57 (1.25–1.96)	**9.1e-05**
Positive	343	1.52 (1.17–1.98)	**0.002**

Bold figures indicate statistically significance

*Abbreviations*: *CST* Cystatins, *GC* Gastric cancer, *HR* Hazard ratio, *CI* Confidence interval

**Table 5:** Stratified analysis of association between CST4 and overall survival in KM plotter GC cohort

**Variables**	**Patients (*n* = 875)**	**HR (95%CI)**	**Log-rank *P*-value**
**Gender**			
Female	236	1.51 (1.06–2.14)	**0.021**
Male	544	1.40 (1.13–1.74)	**0.002**
Missing	95		
**Stage**			
I	67	2.00 (0.73–5.50)	0.170
II	140	1.26 (0.70–2.29)	0.440
III	305	1.68 (1.26–2.25)	**< 0.001**
IV	148	1.52 (1.03–2.23)	**0.033**
Missing	215		
**Lauren classification**			
Intestinal	320	2.09 (1.52–2.89)	**4.3e-06**
Diffuse	241	1.60 (1.14–2.26)	**0.007**
Mixed	32	0.45 (0.15–1.33)	0.140
Missing	282		
**Differentiation**			
Poorly	165	1.35 (0.91–2.01)	0.140
Moderately	67	1.51 (0.78–2.90)	0.210
Well	32	3.21 (1.28–8.09)	0.009
Missing	611		
**Treatment**			
Surgery alone	380	1.51 (1.13–2.01)	**0.005**
5-Fu based adjuvant	152	0.92 (0.65–1.30)	0.630
Other adjuvant	76	1.12 (0.46–2.68)	0.810
Missing	267		
**HER2 status**			
Negative	532	1.29 (1.03–1.62)	**0.025**
Positive	343	1.62 (1.25–2.11)	**< 0.001**

Bold figures indicate statistically significance

*Abbreviations*: *CST* Cystatins, *GC* Gastric cancer, *HR* Hazard ratio, *CI* Confidence interval

### Immunohistochemistry

A total of 115 cases of GC tissues and 20 cases of adjacent tissues with complete clinical data were collected from the Affiliated Hospital of Guangxi Medical University. The demographic and clinical data are shown in Table 6. The results showed that among the clinicopathological parameters of GC in our hospital, tumor stage is associated with patient survival.

**Table 6:** Demographic and clinical data for 115 GC patients

**Variable**	**Patients (*n* = 115)**	**No. of events (%)**	**MST (months)**	**HR (95% CI)**	**Log-rank *P*-value**
Gender					0.687
Male	75	49 (62.7)	50.0	Ref.	
Female	40	27 (60.0)	32.5	1.10 (0.68–1.77)	
Age (years)					0.839
< 60	69	43 (58.0)	45.0	Ref.	
≥ 60	46	33 (67.4)	52.0	1.05 (0.66–1.65)	
Tumor location					0.052
Gastroesophageal junction	12	10 (92.3)	41.5	Ref.	
Gastric body/fundus	31	25 (80.0)	50	0.77 (0.37–1.61)	
Antrum	67	37 (49.3)	61	0.48 (0.24–0.97)	
Missing	5				
**Histologic grade**					0.106
**G2**	17	9 (47.1)	205.0	Ref.	
**G3**	86	59 (64.0)	47.5	1.73 (0.97–3.11)	
Missing	9				
Tumor stage					**< 0.001**
I	21	6 (23.8)	205	Ref.	
II	16	12 (75.0)	47.5	4.82 (1.69–13.72)	
III	32	20 (53.1)	59	3.14 (1.18–8.37)	
IV	45	38 (81.8)	26	7.00 (2.74–17.89)	
Missing	1				
Chemotherapy					0.259
Yes	53	33 (56.6)	55	Ref.	
No	60	42 (66.7)	43.5	1.29 (0.82–2.04)	
Missing	2				

*Abbreviations*: *HR* Hazard ratio, *CI* Confidence interval, *MST* Median survival time

**Fig. 5:**
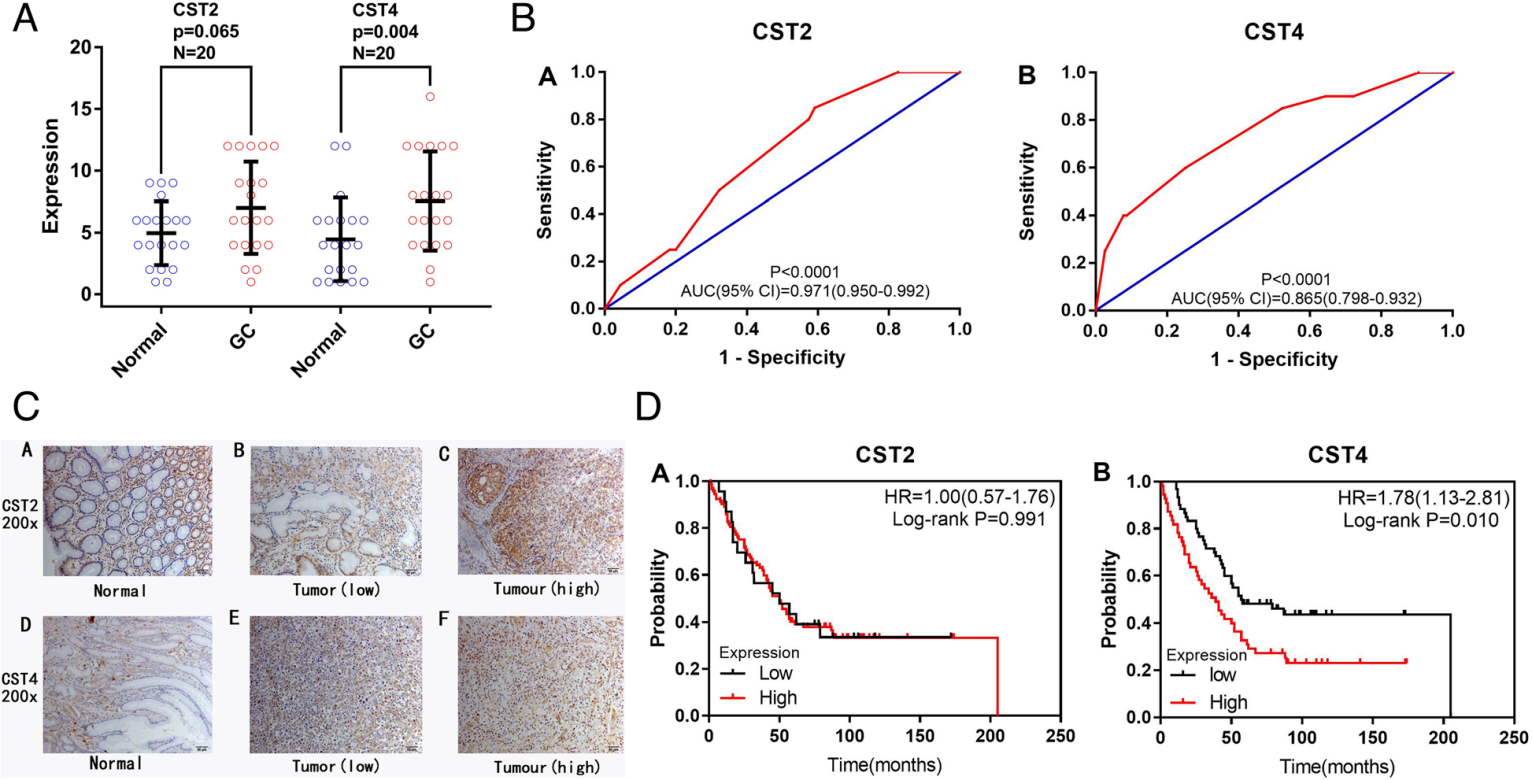
Immunohistochemical analysis. **A** The scatter plot of CST2, CST4 immunohistochemical staining semi-quantitative score of gastric cancer and adjacent normal tissues in affiliated hospital of guangxi medical university. **B** ROC curve of the expression level of CST2, CST4 in affiliated hospital of guangxi medical university. **C** The IHC staining for CST2 and CST4 in adjacent normal tissues and gastric cancer (IHC, ×200). **D** Survival analysis of CST2,CST4 immunohistochemical expression in gastric cancer in affiliated hospital of guangxi medical university. CST, Cystatins

Figure 5A shows the immunohistochemical semi-quantitative score scatter plot of 20 cases of GC and the corresponding adjacent normal tissues. The expression of CST2 and CST4 was higher in GC tissues than in adjacent normal tissues, and the difference of CST4 was statistically significant (*P* < 0.05). The ROC curve of the expression levels of CST2 and CST4 is shown in Fig. 5B, and the difference was statistically significant (*P* < 0.05). Figure 5C shows the immunohistochemical staining images of CST2 and CST4. In the univariate survival analysis, patients with high CST4 immunohistochemical expression in GC tissues had a shorter OS than those with low expression (*P* = 0.010, HR = 1.78, 95% CI = 1.13–2.81, Fig. 5D). The results in Table 6 showed that among the clinicopathological parameters of GC in our immunohistochemistry experiments, tumor stage is associated with OS. Therefore, after adjusting for the tumor stage in the multivariate Cox model survival analysis, the survival time of GC patients with high CST4 immunohistochemical expression was significantly different from that of GC patients with low CST4 immunohistochemical expression (*P* = 0.015, HR = 1.76, 95% CI = 1.76–2.78) (Table 7). In both univariate survival analysis and multivariate Cox model survival analysis, no significant difference was observed in survival time of GC patients with high CST2 immunohistochemical expression in our hospital *(P* > 0.05, Table 7; Fig. 5D).

**Table 7:** Analysis of the association between CST2,CST4 and the risk of death in affiliated hospital of guangxi medical university (*n* = 115)

**Gene expression status**	**Patients (*n* = 351)**	**Events, n (%)**	**MST (days)**	**Crude HR (95% CI)**	**Crude *P*-value**	**Adjusted HR**^**a**^** (95% CI)**	**Adjusted *P*-value**^**a**^
**CST2**					0.991		0.527
Low	23	15(61.1)	50	Ref.		Ref.	
High	92	61(65.0)	50	1.00 (0.57–1.76)		1.20 (0.68–2.12)	
**CST4**					**0.010**		**0.015**
Low	60	34(51.7)	57	Ref.		Ref.	
High	55	42(72.7)	39	1.78 (1.13–2.81)		1.76 (1.11–2.78)	

*Abbreviations*: *GC* Gastric cancer, *CST* Cystatins, *MST* Median survival time, *HR* Hazard ratio, *CI* Confidence interval

^a^Adjusted for tumor stage. Bold figures indicate statistically significance

### Genome-wide co-expression analysis and functional enrichment of type II CST prognostic genes

To determine the underlying mechanism by which CST4 plays a role in GC, we performed a genome-wide co-expression analysis. The regulatory network of CST4 and its co-expressed related genes in GC tissues from the TCGA cohort are shown in Supplemental Fig. S1, Supplemental Data S1. The results showed that 561 genes had positive correlations with CST4 in GC.

The results of GO analysis of CST4 and its co-expressed genes of GC in the TCGA showed that it enriched in focal adhesion, negative regulation of cell proliferation, fascia adherens, cell-matrix adhesion, positive regulation of cell-substrate adhesion, negative regulation of cell migration, etc. (Table 8, Supplemental Data S2). Similarly, KEGG functional enrichment analysis showed that CST4 was enriched in the cGMP-PKG signaling pathway, Calcium signaling pathway, cAMP signaling pathway, and MAPK signaling pathway (Table 9).

**Table 8:** GO term enrichments of co-expressed genes of cystatin 4 in gastric cancer

Term	Count	*P***-**value	Genes
**GO:0005925~focal adhesion**	27	6.47E-05	TGFB1I1, DIXDC1, LPP, AKAP12, CNN1, CSRP2, CSRP1, FLRT1, FLNA, PGM5, FLNC, PIP5K1C, WASF1, PDLIM7, TNS1, SVIL, PPP1R12A, ACTN1, ITGA1, RHOB, NFASC, PALLD, DLC1, CSPG4, FERMT2, VCL, LIMS2
**GO:0008285~negative regulation of cell proliferation**	27	6.71E-05	HDAC4, BTG2, ATP8A2, TGFB1I1, SRF, DPT, GATA3, FGF2, ADRA1A, RERG, GLI3, NACC2, SPEG, KLF10, BCHE, ZBTB16, MAGI2, ITGA1, LDOC1, WNT9A, PHOX2B, AR, ZEB1, BCL6, DLC1, ROR2, FGF10
**GO:0005916~fascia adherens**	4	0.002399344	DES, ACTN1, CTNNA3, VCL
**GO:0007160~cell-matrix adhesion**	9	0.003997901	SRF, ITGA1, ITGA7, NID1, PKD1, FERMT2, VCL, JAM3, TMEM8B
**GO:0010811~positive regulation of cell-substrate adhesion**	6	0.004158018	HACD1, FOXF1, ABI3BP, NPY2R, VWC2, NID1
**GO:0030336~negative regulation of cell migration**	8	0.018321255	IGFBP5, CITED2, SRF, DLC1, TPM1, MAGI2, VCL, RHOB

*Abbreviations*: *GO* Gene ontology

**Table 9:** Kyoto Encyclopedia of Genes and Genomes term enrichments of co-expressed genes of cystatin 4 in gastric cancer

Term	Count	*P*-value	Genes
**hsa04022:cGMP-PKG signaling pathway**	21	4.60E-09	PPP1R12A, SRF, PDE2A, ATP1A4, ATP2B4, ADCY2, ATP1A2, CACNA1C, ADRA1A, SLC8A1, NFATC4, SLC8A2, MYLK, RGS2, PLN, KCNMB1, MRVI1, CALM1, SLC25A4, MYL9, PRKG1
**hsa04020:Calcium signaling pathway**	20	2.04E-07	CHRM2, PDE1C, CAMK2A, ATP2B4, TACR2, ADCY2, CACNA1C, GRPR, ADRA1A, CACNA1H, SLC8A1, RYR3, SLC8A2, MYLK, PLN, NOS1, CALM1, SLC25A4, PLCD4, CAMK2G
**hsa04270:Vascular smooth muscle contraction**	16	3.65E-07	PPP1R12A, PLA2G2C, ADCY2, CACNA1C, ADRA1A, ACTG2, MYLK, ACTA2, MYL6, CALD1, KCNMB1, MRVI1, CALM1, PPP1R12C, MYL9, PRKG1
**hsa04261:Adrenergic signaling in cardiomyocytes**	17	5.95E-07	TPM2, TPM1, CAMK2A, ATP1A4, ATP2B4, CACNA2D3, ADCY2, ATP1A2, CACNA1C, ADRA1A, CACNB2, RPS6KA5, PLN, PPP1R1A, SCN7A, CALM1, CAMK2G
**hsa05414:Dilated cardiomyopathy**	13	1.76E-06	TPM2, TPM1, ITGA1, CACNA2D3, ADCY2, CACNA1C, CACNB2, PLN, DES, SGCD, ITGA7, DMD, SGCG
**hsa05410:Hypertrophic cardiomyopathy (HCM)**	12	5.47E-06	CACNB2, DES, PRKAA2, SGCD, TPM2, TPM1, ITGA1, CACNA2D3, ITGA7, DMD, CACNA1C, SGCG
**hsa04713:Circadian entrainment**	13	6.59E-06	ADCYAP1R1, CAMK2A, ADCY2, CACNA1C, CACNA1H, RYR3, PER1, RPS6KA5, PER3, NOS1, CALM1, CAMK2G, PRKG1
**hsa04921:Oxytocin signaling pathway**	16	8.75E-06	PPP1R12A, PRKAA2, CAMK2A, CACNA2D3, ADCY2, CACNA1C, RYR3, NFATC4, MYLK, CACNB2, RGS2, MYL6, CALM1, CAMK2G, PPP1R12C, MYL9
**hsa05412:Arrhythmogenic right ventricular cardiomyopathy (ARVC)**	10	5.94E-05	CACNB2, DES, SGCD, ITGA1, CACNA2D3, ITGA7, DMD, CTNNA3, CACNA1C, SGCG
**hsa04970:Salivary secretion**	11	8.14E-05	ATP1A4, ATP2B4, ADCY2, ATP1A2, NOS1, CALM1, ADRA1A, CST5, RYR3, PRKG1, CST4
**hsa04024:cAMP signaling pathway**	15	7.23E-04	CHRM2, ADCYAP1R1, PPP1R12A, CAMK2A, ATP1A4, ATP2B4, ADCY2, ATP1A2, CACNA1C, GLI3, PLN, CNGA3, CALM1, CAMK2G, MYL9
**hsa04810:Regulation of actin cytoskeleton**	14	3.66E-03	CHRM2, PPP1R12A, ACTN1, ITGA1, FGF2, MYLK, CFL2, ITGA7, PIP5K1C, WASF1, PPP1R12C, MYL9, VCL, FGF10
**hsa04010:MAPK signaling pathway**	15	6.99E-03	DUSP3, GADD45B, SRF, CACNA2D3, ARRB1, CACNA1C, HSPA2, FGF2, CACNA1H, CACNB2, RPS6KA5, FLNA, MAPT, FLNC, FGF10
**hsa04911:Insulin secretion**	8	7.03E-03	ADCYAP1R1, KCNMB1, CAMK2A, ATP1A4, ADCY2, ATP1A2, CACNA1C, CAMK2G
**hsa04725:Cholinergic synapse**	9	8.95E-03	CHRM2, SLC5A7, CHRNB4, CAMK2A, KCNQ4, ADCY2, CACNA1C, CAMK2G, SLC18A3
**hsa04971:Gastric acid secretion**	7	1.24E-02	CAMK2A, ATP1A4, ADCY2, ATP1A2, CALM1, CAMK2G, MYLK
**hsa04514:Cell adhesion molecules (CAMs)**	10	1.27E-02	NLGN1, NFASC, NLGN4X, NEGR1, NRXN1, LRRC4, NCAM2, LRRC4C, JAM2, JAM3
**hsa04260:Cardiac muscle contraction**	7	1.41E-02	CACNB2, TPM2, TPM1, ATP1A4, CACNA2D3, ATP1A2, CACNA1C
**hsa04925:Aldosterone synthesis and secretion**	7	1.99E-02	PDE2A, CAMK2A, ADCY2, CACNA1C, CALM1, CAMK2G, CACNA1H
**hsa04114:Oocyte meiosis**	8	2.74E-02	AR, CPEB1, PTTG2, CAMK2A, PGR, ADCY2, CALM1, CAMK2G
**hsa04080:Neuroactive ligand-receptor interaction**	14	3.17E-02	CHRM2, ADCYAP1R1, CHRNB4, GRIK5, MLNR, NPY2R, TACR2, GRIK2, GRPR, ADRA1A, GLRB, CNR1, GALR1, NMUR1
**hsa05205:Proteoglycans in cancer**	11	3.91E-02	PPP1R12A, HPSE2, CAMK2A, FLNA, ANK2, WNT9A, FLNC, CAMK2G, ESR1, FGF2, PPP1R12C
**hsa04510:Focal adhesion**	11	4.62E-02	PPP1R12A, ACTN1, ITGA1, CHAD, FLNA, ITGA7, FLNC, PPP1R12C, MYL9, VCL, MYLK
**hsa04922:Glucagon signaling pathway**	7	4.68E-02	PRKAA2, CAMK2A, ADCY2, PYGM, CALM1, ACACB, CAMK2G
**hsa04710:Circadian rhythm**	4	4.76E-02	PER1, PRKAA2, PER3, CRY2
**hsa04530:Tight junction**	6	8.00E-02	ACTN1, MYH11, AMOTL1, MYL9, JAM2, JAM3
**hsa05031:Amphetamine addiction**	5	9.66E-02	MAOB, CAMK2A, CACNA1C, CALM1, CAMK2G
**hsa04720:Long-term potentiation**	5	0.096645635	PPP1R1A, CAMK2A, CACNA1C, CALM1, CAMK2G

*Abbreviations*: *Hsa* Homo sapiens

## Discussion

Cancer development is a multifactorial long-term interaction process, and normal cells undergo multiple steps to transform into malignant cells [25]. Many stages of cancer growth and progression are associated with dysregulation of protease activity [26, 27]. Cysteine proteases are a class of intracellular proteins with a protein-degrading activity that affect a variety of biological processes, including inflammation, immune responses, and cancer [28, 29]. Changes in the expression of cysteine proteases are regulated by their endogenous inhibitor CST [30]. CST can effectively inhibit the activity of cysteine proteases by binding to their active site to form tight equimolar complexes.

The maintenance of the balance between CST and cysteine proteases is crucial to the normal function of biological systems. Once the balance is disrupted, it will cause cell death, leading to tumorigenesis of malignancies [31]. CSTs were originally considered as TPIs; however, this view has changed considerably over the past few decades with various studies targeting the value of CST in tumors. Recently, researchers have revealed that CST affects all stages of cancer progression, including tumor growth, apoptosis, invasion, metastasis, angiogenesis, and antitumor immune responses [32]. In particular, type II CSTs are involved in the metastasis of several types of malignant tumors [12, 33]. However, data regarding the relationship between the expression of type II CST genes and the prognosis of patients with GC are unavailable.

The combination of serum CST4 and DR-70 was reported to further enhance the diagnosis of early colorectal cancer [34]. Recent studies also indicated CST4 was a novel and improved diagnostic marker for colorectal cancer [35]. In the present study, the TCGA database was used to analyze the expression of type II CST genes in GC tissues and adjacent normal tissues. The results showed that the mRNA expression levels of CST1, CST2, and CST4 were higher in GC tissues than in adjacent normal tissues, while the mRNA expression level of CST3 was lower in GC tissues than in adjacent normal tissues. Our immunohistochemical analysis based on the tissues collected from the Affiliated Hospital of Guangxi Medical University also showed that the expression of the CST4 protein was higher in GC tissues than in the adjacent tissues. Dou et al. showed that the mRNA and protein expression of CST4 was significantly upregulated in GC tissues and cell lines, and its sensitivity and specificity for diagnosing GC were 69.0% and 85.6%, respectively [36]. The study of Zhang et al. [37] also supports this conclusion. These studies suggest that CST4 may be a potential novel tumor marker for GC.

Wang et al. [38] indicated that CST4 may be useful in predicting the prognosis of ovarian cancer. In our present study, both univariate survival analysis and multivariate Cox regression survival analysis in the TCGA database showed that the OS of GC patients with high expression of CST2, CST4, and CST5 mRNA was shorter than that of GC patients with low expression of these mRNAs. In the KM plotter online database, the results of survival analysis showed that GC patients with high expression of CST1, CST2, CST3, CST4, CST5, and CST6 had a shorter OS than those with low expression of these genes. The combined results of the TCGA database and the KM plotter online database indicated that CST2 and CST4 may play a role in the prognosis of GC. Accordingly, we screened out CST2 and CST4 for further immunohistochemical experimental validation. In the univariate survival analysis and multivariate Cox model in our study, GC patients with high CST4 immunohistochemical expression had a shorter OS than those with low CST4 expression; this finding was consistent with the results obtained for the TCGA database and the KM plotter online database. In both univariate survival analysis and multivariate Cox model survival analysis, no significant difference was observed in the survival time of patients with high CST2 immunohistochemical expression. According to the results of the nomogram study, the combination of CST2, CST4, and other markers may be considered as a new method to predict the prognosis of GC, and the predictive effect of CST4 was greater than that of age and targeted therapy. Stratified analysis also suggested that CST2 and CST4 have value as an independent prognostic indicator for GC. Taken together, we concluded that GC patients with high expression of CST4 show poor prognosis.

The mechanism by which CST4 plays a role in the occurrence and development of GC has not yet been elucidated. Zhang et al. [37] found that CST4 overexpression promoted the proliferation, migration, and invasion of GC cells in vitro and significantly promoted the tumorigenicity of GC cells in vivo, while silencing CST4 yielded the opposite results. Moreover, silencing CST4 significantly inhibited the migration and invasion ability of GC cell lines and lung metastasis in vivo, while overexpression of CST4 had the opposite effect. It is speculated that the mechanism may be related to the enhancement of the invasiveness of GC by CST4 by regulating the downstream target fibronectin type III domain containing 2 (ELFN2) signaling pathway. To further study the mechanism of CST4 in the development of GC, we used bioinformatics tools, namely genome-wide co-expression analysis, GO term analysis, and KEGG functional enrichment analysis. The genome-wide co-expression analysis of CST4 in GC indicated that CST4 had a co-expression relationship with several genes. The results of GO term analysis showed that CST4 was involved in focal adhesion, negative regulation of cell proliferation, fascia adherens, cell-matrix adhesion, positive regulation of cell-substrate adhesion, and negative regulation of cell migration. Abnormal expression of cell adhesion molecules is often associated with general carcinogenesis and may lead to abnormal proliferation of normal cells [39]. In the present study, the KEGG functional enrichment analysis revealed that CST4 was involved in the regulation of the cGMP-PKG signaling pathway, calcium signaling pathway, cAMP signaling pathway, and MAPK signaling pathway. Activation of the cGMP/PKG signaling pathway promotes the development and progression of GC [40, 41]. The calcium signaling pathway is related to the process of angiogenesis and proliferation of cancer cells [42, 43]. The spatial regulation of cAMP is critical for accurate signal coding [44]. The MAPK signaling pathway plays an important role in controlling cellular processes, including proliferation, differentiation, and apoptosis [45].

The present study has one potential limitation. We used clinical specimens to validate the results obtained by bioinformatics, which largely confirmed the differential expression of CST4 and its significance in the survival and prognosis of patients with GC. However, we were unable to conduct in vivo and in vitro experiments to further confirm the mechanism by which CST4 affects GC occurrence and development. This aspect will be the focus of our future studies.

## Conclusion

In conclusion, this study systematically investigated the relationship between the expression of type II CST genes and OS in patients with GC. As the findings suggest, high expression of CST4 in GC is correlated with a poor prognosis. Therefore, CST4 may be a novel prognostic and predictive indicator for GC.

### Supplementary Information

**Additional file 1: Supplemental Table 1.** Demographic and clinical data for 351 GC patients.

**Additional file 2: Supplemental Figure S1.** Pearson correlation analysis of correlation between TCGA genome-wide expression profile data and CST4.

**Additional file 3： Supplemental Data S1.** CST4 and its co-expressed related genes in GC tissues from the TCGA cohort.

**Additional file 4: Supplemental Data S2.** GO term enrichments of co-expressed genes of CST4 in gastric cancer.

## Data Availability

The datasets generated and analysed during the current study are available in the KM plotter online database (http://kmplot.com/analysis/index.php?p=service&cancer=gastric) and TCGA (https://portal.gdc.cancer.gov). The original contributions presented in the study are included in the article/Supplementary Material. Further inquiries can be directed to the corresponding authors.
