# Supplementary material for: Investigating the prognostic and predictive value of the type II cystatin genes in gastric cancer
Source: BMC Cancer. 2023 Nov 17;23:1122. doi: 10.1186/s12885-023-11550-6 (PMC10657128; doi:10.1186/s12885-023-11550-6)
Supplement: Supplementary file 1 — Additional file 1: Supplemental Table 1. Demographic and clinical data for 351 GC patients. [file 12885_2023_11550_MOESM1_ESM.docx]

SUPPLEMENTAl TABLE 1. Demographic and clinical data for 351 GC patients

| Variable | Patients  (n=351) | No. of  events (%) | MST  (days) | HR (95% CI) | Log-rank  *P*-value |
| --- | --- | --- | --- | --- | --- |
| Gender |  |  |  |  | 0.178 |
| Male | 226 | 100(44.2) | 869 | Ref. |  |
| Female | 125 | 44(35.2) | 1043 | 0.78（0.56-1.10） |  |
| Age (years) |  |  |  |  | **0.022** |
| <60 | 108 | 36(33.3) | 1811 | Ref. |  |
| ≥60 | 240 | 108(45.0) | 766 | 1.54 (1.09–2.18) |  |
| Missing | 3 |  |  |  |  |
| [Tumor](javascript:;) [location](javascript:;) |  |  |  |  | 0.919 |
| Gastroesophageal junction | 84 | 36(42.9) | 792 | Ref. |  |
| Gastric body/fundus | 123 | 50(40.7) | 832 | 0.92(0.60–1.41) |  |
| Antrum | 130 | 52(40.0) | 1043 | 0.94(0.61–1.43) |  |
| Missing | 14 |  |  |  |  |
| Hp infection |  |  |  |  | 0.304 |
| Positive | 18 | 6(33.3) | 1747 | Ref. |  |
| Negative | 143 | 60(42.0) | 1294 | 1.55 (0.67–3.61) |  |
| Missing | 190 |  |  |  |  |
| Histological_type |  |  |  |  | 0.057 |
| Intestinal | 160 | 64(40.0) | 1153 | Ref |  |
| Diffuse Type | 61 | 24(39.3) | 1811 | 1.00（0.63-1.60） |  |
| Signet Ring Type | 11 | 8(72.7) | 387 | 2.52（1.20-5.25） |  |
| Other | 118 | 48(40.7) | 766 | 1.29（0.88-1.88） |  |
| Missing | 1 |  |  |  |  |
| Histologic grade |  |  |  |  | 0.169 |
| G1 | 9 | 2(22.2) | N/A | Ref. |  |
| G2 | 127 | 48(37.8) | 1294 | 1.67 (0.41–6.86) |  |
| G3 | 206 | 90(43.7) | 794 | 2.22 (0.55–9.01) |  |
| Missing | 9 |  |  |  |  |
| MMS |  |  |  |  | 0.225 |
| MSI-H | 240 | 99(41.3) | 832 | Ref. |  |
| MSI-L | 51 | 22(43.1) | 874 | 1.26 (0.79–2.00) |  |
| MMS | 59 | 23(39.0) | 1043 | 0.76 (0.48–1.19) |  |
| Missing | 1 |  |  |  |  |
| Tumor stage |  |  |  |  | **<0.001** |
| I | 47 | 11(23.4) | 2197 | Ref |  |
| II | 109 | 34(31.2) | 1686 | 1.61（0.81-3.18） |  |
| III | 147 | 69(46.9) | 779 | 2.44（1.29-4.61） |  |
| IV | 35 | 22(62.9) | 476 | 3.79（1.84-7.82） |  |
| Missing | 13 |  |  |  |  |
| Cancer status |  |  |  |  | **<0.001** |
| Tumor free | 206 | 35(17.0) | N/A | Ref. |  |
| Withtumor | 118 | 86(72.9) | 507 | 5.53 (3.70–8.26) |  |
| Missing | 27 |  |  |  |  |
| Residual_tumor |  |  |  |  | **<0.001** |
| R2 | 14 | 9 (64.3) | 451 | Ref. |  |
| R1 | 14 | 12(85.7) | 274 | 3.83 (1.59–9.23) |  |
| R0 | 287 | 100(34.8) | 1407 | 0.53 (0.27–1.06) |  |
| Missing | 36 |  |  |  |  |
| Radiotherapy |  |  |  |  | **<0.001** |
| Yes | 62 | 19(30.6) | N/A | Ref. |  |
| No | 266 | 116(43.6) | 779 | 2.32 (1.42–3.80) |  |
| Missing | 23 |  |  |  |  |
| Target Therapy |  |  |  |  | **0.023** |
| Yes | 151 | 56(37.1) | 1294 | Ref. |  |
| No | 175 | 78(44.6) | 766 | 1.49 (1.06–2.10) |  |
| Missing | 25 |  |  |  |  |

Notes: Bold figures indicate statistically significance.

Abbreviations: GC, gastric cancer; MST, median survival time; HR, hazard ratio; CI, confidence interval; N/A, not available; HP, Helicobacter pylori (H. pylori)
