# Supplementary figures and images for: Investigating the prognostic and predictive value of the type II cystatin genes in gastric cancer
Source: BMC Cancer. 2023 Nov 17;23:1122. doi: 10.1186/s12885-023-11550-6 (PMC10657128; doi:10.1186/s12885-023-11550-6)

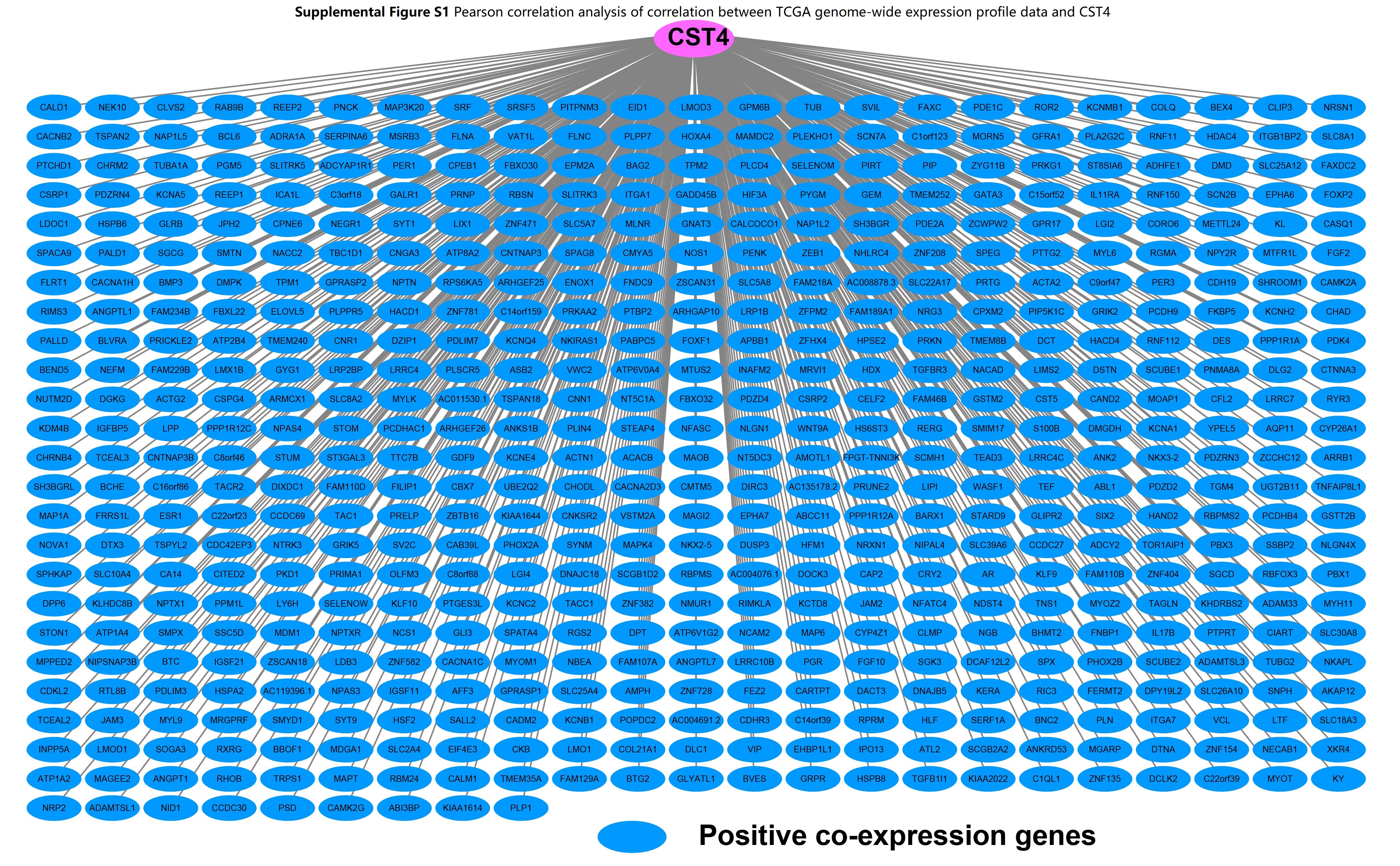

Supplement: Supplementary file 2 — Additional file 2: Supplemental Figure S1. Pearson correlation analysis of correlation between TCGA genome-wide expression profile data and CST4. [file 12885_2023_11550_MOESM2_ESM.jpg]
